# Intravascular Large B-Cell Lymphoma: A Review with a Focus on the Prognostic Value of Skin Involvement

**DOI:** 10.3390/curroncol29050237

**Published:** 2022-04-19

**Authors:** Thomas Breakell, Heidi Waibel, Stefan Schliep, Barbara Ferstl, Michael Erdmann, Carola Berking, Markus V. Heppt

**Affiliations:** 1 Department of Dermatology, Universitätsklinikum Erlangen, Friedrich-Alexander-Universität Erlangen-Nürnberg, Ulmenweg 18, 91054 Erlangen, Germany; breakell.tom@gmail.com (T.B.); stefan.schliep@uk-erlangen.de (S.S.); michael.erdmann@uk-erlangen.de (M.E.); carola.berking@uk-erlangen.de (C.B.); 2 Comprehensive Cancer Center Erlangen-European Metropolitan Area of Nuremberg (CCC ER-EMN), 91054 Erlangen, Germany; 3 Department of Internal Medicine 5, Universitätsklinikum Erlangen, Friedrich-Alexander-Universität Erlangen-Nürnberg, Ulmenweg 18, 91054 Erlangen, Germany; heidi.waibel@uk-erlangen.de (H.W.); barbara.ferstl@uk-erlangen.de (B.F.)

**Keywords:** IVLBCL, intravascular large B-cell lymphoma, R-CHOP, prognostic model, dermato-oncology, skin cancer

## Abstract

Intravascular large B-cell lymphoma (IVLBCL) is an aggressive Non-Hodgkin lymphoma (NHL) characterised by the presence of neoplastic lymphoid cells within small- and medium-sized blood vessels. According to the clinical presentation, the current WHO classification distinguishes the ‘classic’ (formerly ‘Western’) from a hemophagocytic syndrome-associated (formerly ‘Asian’) variant. A third ‘cutaneous’ variant has been proposed, characterised by a good prognosis and unique clinical features. While laboratory findings can hint at diagnosis, symptoms are rather nonspecific, and deep skin biopsy supported by further measures such as bone marrow aspiration and positron emission tomography-computed tomography scanning is needed to make a definite diagnosis. Treatment is comprised of anthracycline-based chemotherapy supplemented with rituximab and central nervous system prophylaxis. While there are various prognostic models for NHL, only one is specific to IVLBCL, which does not sufficiently represent some patient groups, especially regarding the lack of differentiation within the patient collective with skin involvement. This underlines the necessity for the establishment of further prognostic models in particular for IVLBCL patients with cutaneous manifestations.

## 1. Epidemiology and Aetiology

Intravascular large B-cell lymphoma (IVLBCL) is a rare type of extranodal Non-Hodgkin lymphoma (NHL) with largely unknown pathogenesis. IVLBCL is characterised by intravascular proliferation of neoplastic lymphoid cells, leading to occlusion of medium- and small-sized vessels and subsequent organ dysfunction. Major parenchymatous infiltration by tumour cells does not usually occur. Sexes are equally affected. The median age at diagnosis is between 60 and 70 years, but the exact incidence of IVLBCL remains unknown [1,2,3]. It is estimated to be lower than 0.5 cases per million inhabitants underlining its orphan nature [4]. Older terms for the disease are ‘angiotropic large cell lymphoma’ and ‘intravascular lymphomatosis’ [5,6].

Very little is known about the genetics of the disease. Diverse translocations are known to contribute to the pathogenesis of IVLBCL, including B-cell lymphoma 2 (Bcl2) and B-cell lymphoma 6 (Bcl6) genes, the overexpression of the former having been shown to play a role in the inhibition of apoptosis and subsequent lymphoma development. Specifically, in IVLBCL, Bcl6 positivity was found in approximately 25% of cases with immunohistochemistry [7,8]. Structural variants of the programmed death-ligand 1 (PD-L1) and programmed death-ligand 2 (PD-L2) genes additionally hint at immune evasion being involved in the aetiology of the disease. A recent study detected genetic PD-L1 and PD-L2 rearrangements at a high frequency in IVLBCL, as well as structural variants correlating with overexpression of both genes. These results suggest that escaping immune surveillance is involved in the pathogenesis and raises the possibility that immune checkpoint blocking agents show antitumour efficacy in IVLBCL [9].

## 2. Extracutaneous Clinical Presentation

IVLBCL is an aggressive disease, frequently disseminating. Common findings include B symptoms, anemia, and elevated lactate dehydrogenase (LDH) levels [3]. Nevertheless, the clinical manifestations are highly variable and non-specific in most cases. Interestingly, the symptoms differ depending on the geographic origin of the patients where two major clinical variants can be discerned. Historically, the terms ‘Western’ and ‘Asian’ were used to account for these differences in clinical presentation and are still used in literature. However, the symptoms may vary and globalisation, travel behaviours, and increasing mobility have challenged the usage of these terms. To account for this, the recent WHO classification does not make a distinction about geographic origin anymore but suggests the alternative terms ‘classic’ and ‘hemophagocytic syndrome-associated’ according to the clinical presentation.

The classic variant comes along with general weakness, recurring fevers, night sweats, significant weight loss and rapidly progressing neurological symptoms. In contrast to the hemophagocytic syndrome-associated variant, bone marrow, liver, and spleen are less commonly affected, whereas the involvement of the central nervous system (CNS) is common. The neurological symptoms are varied and may include general cognitive impairment with dementia, peripheral neuropathy, focal deficits due to ischemic lesions, or signs of myelopathy due to venous congestion [10]. A recent case was reported with brain involvement mimicking progressive multifocal leukoencephalopathy with a rapidly progressive neurological deterioration [11]. A similar case was reported in a 54-year-old man who suffered from IVLBCL affecting multiple cranial nerves but did not show any other symptoms [12]. In these cases of distinct but non-specific neurological symptoms but a lack of involvement of bone marrow, skin, or lymph nodes, the intravital diagnosis of IVLBCL is difficult. This reflects the fact that 50% of the diagnoses of the Western variant are made only post-mortem, following an autopsy [1]. In such cases of unclear symptoms, random skin biopsies of clinically unaffected skin can be helpful to make the correct diagnosis [13].

The hemophagocytic syndrome-associated variant has a more lymphoma-typical clinical image, while neurological or cutaneous symptoms are less common. In contrast, involvement of the bone marrow, spleen, and liver are frequently observed, and the patients present with hepatomegaly, splenomegaly, or recurring fevers. Non-specific symptoms of this variant include loss of appetite, fatigue, and chills while lymphadenopathy is usually absent in the majority of cases. The hemophagocytic syndrome-associated variant, per definition, includes haemophagocytic syndrome or haemophagocytic histiocytosis, which manifests as cytopenia and usually portends a poor prognosis [3,14,15,16].

In both variants, the leading symptom is a fever of unknown origin with rapid clinical deterioration in the absence of evidence for infection without prominent lymphadenopathy. Furthermore, both variants can have overlapping features and the distinction between those main types is not clear-cut in all cases. A recent case of a Chinese patient, for example, showed both neurological involvement and hemophagocytic syndrome [17] (Table 1). Rather unusual and rare presentations of IVLBCL reported in the literature include refractory acidosis [18], spontaneous tumour lysis syndrome, dyspnoea with subsequent lactic acidosis, pulmonary embolism, affection of the cervix uteri [19], renal involvement with elevated serum anti-neutrophil cytoplasmic antibody (ANCA) titres [20], primary prostatic [21], or uveal [22] involvement.

These various clinical presentations show that IVLBCL can affect virtually any organ system, depending on the location of the vasculopathy resulting from the intraluminal proliferation of neoplastic lymphoid cells. Accordingly, the differential diagnosis is exhaustive, leading to a massive delay in the correct diagnosis of IVLBCL in many cases.

## 3. Skin Findings

Along with the CNS, the skin is the most frequently involved organ in IVLBCL. Depending on the study and collective, cutaneous findings were the main clinical presentation in 17-39% of patients. The skin was the only detectable site of disease in two-thirds of cases in one study, with a striking predominance of females and a good performance status in this group. This clinical presentation with manifestations limited to the skin is sometimes referred to as a ‘cutaneous variant’. Notably, survival depended strongly on the number of cutaneous lesions. While patients with only one lesion survived in the long term, most patients with multiple skin manifestations suffered a relapse within a year of treatment and showed a worse outcome. Superior survival of the cases with skin involvement is hypothesised to be due to facilitated and hence timelier diagnosis [3]. It remains to be elucidated whether this is the main factor responsible for better performance of patients with skin manifestations or if it is due to other skin-resident factors.

**Figure 1:**
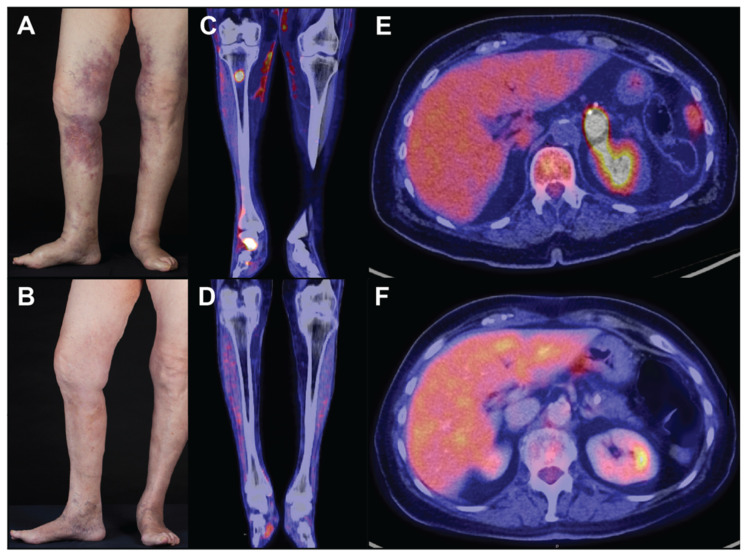
Macroscopic findings in IVLBCL: (**A**,**B**) cutaneous lesions before (**A**) and after (**B**) two cycles of R-CHOP; (**C**–**F**) PET-CT scans of cutaneous and osseous lesions of the legs before (**C**) and after (**D**) six cycles of R-CHOP. Adrenal gland lesion before (**E**) and after (**F**) six cycles of R-CHOP. PET-CT scanning showed increased uptake of fluorodeoxyglucose (^18^F-FDG) in the subcutaneous tissue of both legs, the right tibia, various osseous structures of the right foot (maximal standardised uptake value (SUVmax) = 3.0–21.5), and a nodular lesion in the left adrenal gland (SUVmax = 31).

Cutaneous symptoms are more common in the Western variant and have a preponderance for females [28]. The clinical image encompasses erythematous patches and plaques, frequently affecting the lower extremities or trunk. The findings are usually accompanied by general malaise and recurrent fevers. The authors saw a case of a 77-year-old Caucasian female patient presenting with a three-month history of painful lividly discoloured contusiform nodular lesions on both thighs and lower legs (Figure 1A,B). Abdominal and lumbar back pain had initially raised the suspicion of a rheumatologic condition and led to extensive imaging showing no specific pathologies. Additionally, chronic inflammatory bowel diseases had been ruled out, as the cutaneous manifestation of Crohn’s disease can have a similar clinical presentation. Skin lesions of IVLBCL can be accompanied by pain, telangiectasia, and significant oedema adjacent to the lesions. The latter two features should be distinguished from Pseudo–Kaposi sarcoma possibly observed on the lower legs in a background of chronic venous insufficiency of the elderly. Contusiform lesions of IVLBCL must not be confused with erythema nodosum, traumatic panniculitis, or deep-seated hematoma. Other differentials may include lupus profundus, systemic vasculitis, alpha-1-antitrypsin deficiency, subcutaneous panniculitis-like T-cell lymphoma, eosinophilic fasciitis, autoinflammatory syndromes, or reactive angioendotheliomatosis. (Table 2).

## 4. Diagnosis and Pathological Findings

**Figure 2:**
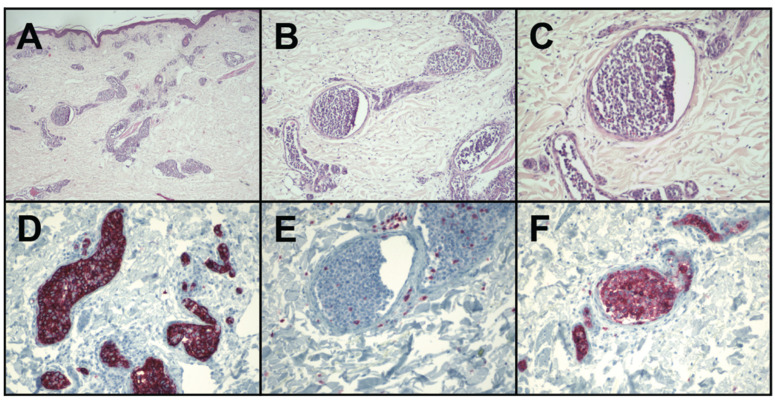
Microscopic findings in IVLBCL. Haematoxylin and eosin stain of deep skin biopsy at 4-fold (**A**), 10-fold (**B**), and 20-fold magnification (**C**); immunohistological skin biopsy stains for CD20 (**D**), CD3 (**E**), and CD79a (**F**). A skin biopsy taken from the left thigh of a patient with IVLBCL showed normal epidermis with compact cornification. The entire dermis and subcutis showed intravasally located large atypical plasmacytoid lymphocytes. Perivascularly, small chromatin-dense lymphocytes were found. Immunohistochemistry revealed positive staining of intravascular lymphocytes for CD20, CD45, CD79a, Bcl-2, and partially positive staining for CD10 and Bcl-6. Few diffusely dispersed small CD3^+^ lymphocytes were also found.

Due to the rather non-specific clinical presentation, the diagnosis of IVLBCL requires histopathologic evaluation of deep skin biopsies [28]. Biopsies from both affected skin and random areas (i.e., appearing clinically unaffected) can be diagnostically relevant, with some authors recommending at least three different harvest sites [13,29,30,31,32,33]. The histopathologic image is characteristic and shows aggregates of large atypical lymphoid or plasmacytoid cells within the lumen of small- and medium-sized blood vessels, mostly in capillaries and post-capillary venules [14]. The morphology of the lymphoid cells varies. They usually have the phenotype of neoplastic B cells, with large and abundant cytoplasm, vesicular nuclei, and prominent nucleoli (Figure 2A–C). The proliferation index of the cells is high. The affected vessels can be infrequent and dispersed throughout the dermis and the subcutaneous fat tissue is often intermingled with unaffected areas. Thus, obtaining a sufficiently deep and large biopsy is critical to capture blood vessels containing affected lymphocytes [28]. The biopsy of pre-existing senile haemangiomas may enhance the likelihood to capture affected vessels and thus increase the sensitivity in case they are present.

In the vast majority of reported cases, there is no dermal or epidermal infiltration of the lymphocytes, and aggregates of the tumour cells are confined to vascular spaces. However, reactive perivascular infiltrates composed of small lymphocytes with dense nuclear chromatin and plasma cells may accompany the intravascular proliferation of the lymphocytes [28]. The lumen of the affected vessel may be partially or completely occluded by atypical lymphocytes.

To differentiate IVLBCL from other T-cell or natural killer (NK) cell lymphomas, immunophenotyping of the intravascular lymphocytes is necessary [34,35]. The intravascular cells usually express CD45, CD20, the surface marker of B cells, and CD79a, a B-cell antigen receptor marker [36,37], but are not reactive to CD3 (Figure 2D–F). The cells have a high Ki-67 proliferation index, which was of negative prognostic value in one retrospective case review [38]. The large atypical cells stain positive for Bcl-2 and multiple myeloma 1 (MUM1) and variably for Bcl-6 and CD10. Monoclonality was reported in some cases but is of limited diagnostic value in our experience [39].

In rare cases with a T-cell or NK cell phenotype, neoplastic cells expressing CD3, granzyme B but not CD20, CD4, CD8, CD5 were proposed [40,41,42]. However, these cases showed an unusual presentation and were almost exclusively reported in Chinese patients. In all these reports, evidence for Epstein–Barr virus infection was found [34,35].

Histologically, the correct diagnosis relies on the recognition and correct interpretation of atypical intravascular lymphoid cells, confirmed by immunohistochemistry (Figure 2). The pathological differential diagnosis includes other metastatic neoplasms, other lymphomas with a large-cell appearance, reactive angioendotheliomatosis, or reactive intravascular histiocytosis, where cells express CD68 in the absence of B-cell markers. Benign intravascular lymphoproliferation with CD30^+^ positive cells can histologically mimic IVLBCL but are exceedingly rare [43,44,45,46,47].

## 5. Pretreatment Evaluations and Treatment

After the histopathologic diagnosis, the pretreatment evaluations should follow the guidelines for advanced stage NHL with diffuse large B-cell lymphoma. The laboratory workup includes a complete blood count with differential blood count, serum LDH, serum albumin, renal and liver panel, and infection serology for hepatitis B and C and human immunodeficiency virus (HIV). A bone marrow biopsy is recommended to assess bone involvement, although negative results do not exclude IVLBCL. The performance of a lumbar puncture to assess leptomeningeal involvement as a routine measure is advisable, as the CNS is commonly affected in IVLBCL, in particular in the classic variant.

Imaging is of paramount importance to determine the affected organ systems and body sites. The modality of choice is fluorodeoxyglucose (^18^F) (^18^F-FDG) positron emission tomography-computed tomography (PET-CT), as IVLBCL is highly FDG-avid, and affected sites will be identified with high sensitivity. Furthermore, the intensity of FDG uptake can indicate the response to treatment (Figure 1C–F) [48,49].

## 6. Treatment and Follow-Up

IVLBCL is treated with chemotherapy using the CHOP regimen comprised of cyclophosphamide, doxorubicin, vincristine (Oncovin^®^), and prednisone combined with rituximab (R-CHOP), the addition of which results in superior response and survival [14,50,51]. This combined treatment yields response rates >60%, subsequent 3-year overall survival (3Y-OS) >30% and possible long-term survival [1,3,13,14,23,52,53,54].

CNS involvement is common in IVLBCL, occurring in 30–40% of patients at diagnosis and presenting in an additional 25% during follow-up [26]. As the R-CHOP regimen does not cross the blood–brain barrier, additional CNS-targeted therapy is warranted. This includes both prophylaxis and treatment, including intrathecal chemotherapy, systemic methotrexate, and escalating measures such as radiation and spinal cord decompression in case of leptomeningeal disease [55,56,57,58].

## 7. Prognostication

IVLBCL is an aggressive and often rapidly progressing form of cancer. The oncologic outcome has been improved by the addition of targeted antibody therapy to traditional chemotherapy regimens. Nevertheless, IVLBCL is a lethal condition in most cases.

Several prognostic models for the classification of lymphomas have been established: The International Prognostic Index (IPI) [59] is the primary clinical tool for outcome prediction of aggressive NHL lymphomas and determines negative prognostic factors to be the following:

- Age > 60;
- Ann-Arbor stage III/IV disease;
- Elevated LDH level;
- Eastern Cooperative Oncology Group (ECOG) performance status [60] ≥ 2;
- >One extranodal site of disease.

Out of the resulting groups, 5-year overall survival (5Y-OS) rates range from 26% to 73% [61].

Another prognostic model, focusing on elderly patients with large B-cell lymphoma treated with R-CHOP additionally identified age >75 years, serum albumin concentration of <3.7 g/dL and a Charlson Comorbidity Index (CCI) ≥ 3 [62] to be—independently of the IPI—associated with worse overall and progression-free survival [63].

Moreover, increased serum levels of soluble IL-2 receptor (sIL-2R) (≥2000 IU/mL) have been shown to result in a reduced 5Y-OS in large B-cell lymphoma patients treated with R-CHOP—namely, 54% versus 89% [64]. For IVLBCL specifically, age >70 years, CNS involvement, and LDH levels ≥700 IU/L were all associated with shorter survival [65].

Independently of these negative predictors, in multiple studies, the presence of cutaneous manifestation was consistently associated with a significant increase in overall survival—namely, an improved average 3Y-OS of 56% versus 22%. Remarkably, in one study, all patients with skin manifestations were younger women with normal leukocyte and platelet counts and were less likely to have B symptoms and bone marrow infiltration [3,14]. However, previous studies and classifications differentiated mainly between the categories ‘cutaneous involvement’ and ‘no cutaneous involvement’, which overlooks the fact that many patients have both visceral and skin manifestations. In future classifications, the separation into three categories, i.e., ‘cutaneous involvement only’ (also referred to as cutaneous variant), ‘cutaneous and further visceral involvement’ and ‘no cutaneous involvement’ could be beneficial to more accurately estimate prognosis, especially as this differentiation has not yet been successfully achieved. While the prognosis for the cutaneous variant is consistently more favourable in previous reports, the outcome of the patients with both cutaneous and visceral manifestations is less clear. The authors recently saw a case of a patient with symptoms of the classical variant and both skin and CNS involvement. In this particular case, cutaneous symptoms were the only positive versus various negative predictors; however, the patient showed long-term remission and survival even without CNS prophylaxis with methotrexate, a treatment shown to reduce the incidence of CNS recurrence in patients treated with R-CHOP [58]. This raises the question of whether patients with cutaneous involvement in fact require CNS prophylaxis even if unfavourable prognostic factors are present. Additionally, in the reported case, the course of the disease was considerably more favourable than anticipated, despite fulfilling many negative predictors and the positive predictor of cutaneous involvement being weakened by the occurrence of multiple skin lesions, demonstrating that this patient was not adequately represented within any of the published prognostic models. We concluded that cutaneous involvement might be a stronger prognostic indicator than previously assumed when weighed against others. Nevertheless, this case observation must not lead to over-interpretation and over-reliance on cutaneous manifestations as a positive prognostic factor, in particular when other prognostically unfavourable factors are present.

These considerations highlight that, in addition to the predominantly clinical indicators, new advances using molecular technologies to improve early diagnosis, prognostic accuracy and therapy are currently being established including mutational analysis using genome sequencing of cell-free deoxyribonucleic acid (DNA) [66]. This could be beneficial when combined with the current practice of random skin biopsies that carry the risk of frequent false negatives, the sensitivity merely being 77.8% [32].

Additionally, testing for tumour cell expression of PD-L1 for IVLBCL is being investigated. The presence of PD-L1 resulted in worse overall survival [9,67] and could represent a rationale for immunotherapy using checkpoint inhibitors in refractory cases. Strikingly, this is the opposite effect positive PD-L1 expression has in metastasised melanoma, where it is associated with increased overall survival [68].

In conclusion, skin findings present important clues for a timely diagnosis of IVLBCL; hence further integration of stratified cutaneous symptoms into prognostic models is warranted.

## Figures and Tables

**Table 1:** Comparison of the two main clinical variants of IVLBCL [2,3,14,15,23,24,25,26,27].

	Classic Variant	Hemophagocytic Syndrome-Associated Variant
Neurological symptoms (CNS involvement)	39–76%, rapidly progressing	27%
Cutaneous symptoms	17–39%	5%
Bone marrow involvement	32%	75%
Splenic involvement	26%	67%
Hepatic involvement	26%	55%
Hemophagocytic syndrome	not present	present
Common findings: - Clinical - Laboratory	B symptoms LDH ↑, sedimentation rate and C-reactive protein (CRP) ↑, anemia	
Differing findings:		
- Thrombocytopenia - Hypoalbuminemia	29% 18%	76% 84%

**Table 2:** Differential diagnosis of cutaneous findings in IVLBCL.

Morphologic Feature	Differential Diagnosis
Erythematous nodule without epidermal involvement	Primary B-cell lymphoma of the skin Pseudolymphoma Cutaneous manifestation of Crohn’s disease
Livid patch/nodule with telangiectasia on lower extremity	Thrombophlebitis Chronic venous insufficiency Acroangiodermatitis (Pseudo–Kaposi sarcoma) Kaposi sarcoma Haemangioma
Painful subcutaneous plaques with erythema and induration	Lupus panniculitis Subcutaneous panniculitis-like T-cell lymphoma Vasculitis (medium-sized vessels) Angioendotheliomatosis Panniculitis of alpha-1-antitrypsin deficiency Eosinophilic fasciitis
Contusiform rash on lower legs	Erythema nodosum Traumatic panniculitis Haematoma
Migrating/Figurate erythema	Autoinflammatory syndromes

## Data Availability

The data presented in this study are available on request from the corresponding author.

## Abbreviations

ANCA	Anti-neutrophil cytoplasmic antibody
Bcl-2	B-cell lymphoma 2
Bcl-6	B-cell lymphoma 6
CCI	Charlson Comorbidity Index
CD	Cluster of differentiation
CNS	Central nervous system
CRP	C-reactive protein
DNA	Deoxyribonucleic acid
ECOG	Eastern Cooperative Oncology Group
HIV	Human immunodeficiency virus
IPI	International Prognostic Index
IVLBCL	Intravascular large B-cell lymphoma
LDH	Lactate dehydrogenase
LEF-1	Lymphoid enhancer-binding factor 1
MUM1	Multiple myeloma 1
NHL	Non-Hodgkin’s lymphoma
NK cell	Natural killer cell
PD-L1	Programmed death-ligand 1
PD-L2	Programmed death-ligand 2
PET-CT	Positron emission tomography-computed tomography
sIL-2R	Soluble IL-2 receptor
SUVmax	Maximal standardised uptake value
^18^F-FDG	Fluorodeoxyglucose (^18^F)
3Y-OS	Three-year overall survival
5Y-OS	Five-year overall survival
